# Gut Microbes Regulate Innate Immunity and Epilepsy

**DOI:** 10.3389/fnins.2022.870197

**Published:** 2022-06-01

**Authors:** Linhai Zhang, Shuang Li, Zhenzhen Tai, Changyin Yu, Zucai Xu

**Affiliations:** ^1^Department of Neurology, Affiliated Hospital of Zunyi Medical University, Zunyi, China; ^2^The Collaborative Innovation Center of Tissue Damage Repair and Regeneration Medicine of Zunyi Medical University, Zunyi, China

**Keywords:** epilepsy, innate immunity, central nervous system, gut–brain axis, microorganisms

## Abstract

Epilepsy is a common chronic brain disease. There are many clinical methods to control epileptic seizures, such as anti-seizure medications (ASMs) or surgical removal of epileptogenic lesions. However, the pathophysiology of epilepsy is still unknown, making it difficult to control or prevent it. The host’s immune system monitors gut microbes, interacts with microbes through pattern recognition receptors such as Toll-like receptors (TLRs) and NOD-like receptors (NLRs) expressed by innate immune cells, and activates immune responses in the body to kill pathogens and balance the relationship between microbes and host. In addition, inflammatory responses induced by the innate immune system are seen in animal models of epilepsy and temporal lobe epilepsy brain tissue to combat pathogens or injuries. This review summarizes the potential relationship between gut microbes, innate immunity, and epilepsy based on recent research to provide more hints for researchers to explore this field further.

## Introduction

As a common chronic neurological disorder, epilepsy is characterized by susceptibility to epileptic seizures and associated cognitive and psychological impairments (Devinsky et al., 2018). According to a meta-analysis, the annual incidence of epilepsy is approximately67.77 per 100,000 persons; age and gender have less effect on the incidence; the incidence is quite high in low- and middle-income nations (Fiest et al., 2017). Recently, intestinal microbial composition, metabolites, and synthetic functions in various neurological diseases have been gradually understood. The regulatory role of intestinal microbes has been found in neuropsychiatric diseases such as Alzheimer’s disease (AD), Parkinson’s disease (PD), and autism spectrum disorder (ASD) (Sharon et al., 2016). Epilepsy is a disabling neurological disorder, and its specific pathogenesis is still unclear. Research into the regulation of brain function by gut signals may help elucidate epilepsy pathogenesis, drug resistance, or potential therapeutic targets. Innate immunity is an important bridge linking vertebrate and invertebrate immune recognition in the long evolution of organisms (Litman and Cooper, 2007). Cellular pattern recognition receptors recognize highly conserved pathogen-associated molecular patterns (PAMPs) produced only by microorganisms to distinguish foreign tissues (Kaur and Secord, 2019). Some studies have pointed out that intestinal dysbiosis can induce peripheral inflammation and central nervous system (CNS) inflammation (or sterile inflammation) by inducing the innate immune system to produce cytokines (Levy et al., 2017). Although the brain is considered an immune-privileged site, there has been a steady stream of research on neuroinflammation and epilepsy (Vezzani et al., 2011). Neuroinflammation is often triggered by danger signals, including endogenous injury or infection, that rapidly activate pattern recognition receptors (PRRs) expressed by innate immune cells, altering seizure thresholds (Vezzani et al., 2016). This review summarizes the potential relationship between gut microbes, innate immunity, and epilepsy to provide more hints for future studies.

## Possible Mechanisms by Which Intestinal Microbes Are Involved in Epilepsy

### Intercommunication Between Intestinal Microbes and the Central Nervous System

There are rich connections between the CNS and intestinal microbes. Research has shown that intestinal microorganisms can modify the CNS, affecting behavior, mood, cognition, and even causing anxiety and depression. The gut–brain axis refers to the interaction between the gut microbes and the CNS, involving multiple systems such as nerves and endocrine (Collins et al., 2012; Holmes et al., 2012, 2020; Nicholson et al., 2012; Montiel-Castro et al., 2013). Intercommunication between intestinal microbes and the CNS involves two neuroanatomical pathways, and one is the direct exchange of information between the brain and the gut, including the vagus nerve in the spinal cord and the autonomic nervous system. The other is a bidirectional communication in the spinal cord through the enteric nervous system, autonomic nervous system, and vagus nerve of the gut (Wang and Wang, 2016).

Although the structure of the microorganism is relatively stable, factors including infection, diet, or genetics can also affect the intestinal flora, resulting in the growth of pathogenic bacteria, the loss of normal commensal bacteria, and the decline in diversity (Levy et al., 2017), which is called dysbiosis, and can further lead to diseases such as obesity (Gérard, 2016), autoimmune disease (Knip and Siljander, 2016), neurological disorders (Tremlett et al., 2017), and inflammatory bowel disease (Wlodarska et al., 2015). A study showed that using mass spectrometry to detect chemicals in the peripheral circulation in germ-free (GF) mice found that the synthesis of most substances depends on the gut microbiome (Wikoff et al., 2009). Substances produced by intestinal microbial metabolism can act on the CNS. For example, short-chain fatty acids (SCFAs) are the final product produced by microorganisms in the lower intestinal tract through fermentation, including acetic acid, propionic acid, and butyric acid (Martin-Gallausiaux et al., 2021), are important substrates for keeping the integrity of the epithelial barrier (Morrison and Preston, 2016), and are also involved in regulating human immune function and exerting anti-inflammatory effects (Martin-Gallausiaux et al., 2021). SCFAs can also penetrate the gut–blood barrier and the blood–brain barrier (BBB) (Macfabe, 2012), alter neurotransmitter and hormone concentrations (Alexander et al., 2019), reduce the permeability of the BBB (Braniste et al., 2014), and regulate the formation and function of microglia (Hu et al., 2020).

In addition, entheogenic GABA, 5-HT, can affect microglial activation in the brain (Abdel-Haq et al., 2019). Diaz Heijtz et al. (2011) showed that GF mice exhibited less anxiety-like behavior than conventional mice fed in a specific pathogen-free (SPF) environment. However, after the adult GF mice were transferred to the SPF environment, there was no significant increase or decrease in anxiety-like behavior, but the anxiety-like behavior of their offspring returned to normal (Diaz Heijtz et al., 2011). It has also been shown that ACTH and corticosterone levels under restraint stress are different in GF mice and SPF mice (Sudo et al., 2004). All of these studies illustrate the ability of gut microbes to regulate behavior.

Although the BBB separates the brain and peripheral tissues, gut microbiota can influence CNS function (Bercik et al., 2011; Diaz Heijtz et al., 2011). By comparing GF and SPF rats, Erny et al. (2015) showed that gut microbiota could affect microglial function, including reducing cell-activated gene transcription and increasing transcriptional repressor expression, and Mosher and Wyss-Coray (2015) observed that entheogenic SCFAs can promote microglial maturation and related gene expression in SPF mice. Microglia are located in the CNS and are the primary immune cells in the brain, but they are closely related to the mononuclear macrophage system (Erny et al., 2015) and can generate immune responses to peripheral inflammation. Combrinck et al. (2002) used an intraperitoneal injection of lipopolysaccharide (LPS) to induce peripheral systemic inflammation and observed further activation of activated microglia in the brain in animals with chronic inflammation, suggesting that there is an interaction between peripheral systemic inflammation and the CNS.

Gut microbial surface substances and their metabolites can positively or negatively affect the CNS through the peripheral circulation or enteric nervous system, and microbial-derived neurotransmitters have regulatory effects on neurons or nerve cells after entering the brain. The metabolites can not only play an anti-inflammatory effect and protect the BBB but also play a pro-inflammatory effect, activate microglia to secrete inflammatory mediators, and then damage the BBB, leading to leakage.

### General Characteristics of the Gut Microbiota in Epileptic and Non-epileptic Patients

Gut microbes are a microbial ecosystem that exists in the human gastrointestinal tract. The average adult’s gut microbes weigh as much as the human brain, and these microbes encode genes that are more than 100 times more abundant than the human genome (Dinan et al., 2015), and there are more than 10^14^ kinds of microorganisms. In addition to bacteria, archaea, viruses, bacteriophages, yeasts, and fungi coexist in the gut (Cani, 2018). This complex microbial population is increasingly becoming an important role in affecting human health and is closely related to the stability of the human body’s internal environment. For example, (1) the formation of a bacterial barrier in the gut prevents pathogenic bacteria from penetrating the intestinal mucosal barrier and invading the host (Chopyk and Grakoui, 2020), (2) the synthesis of nutrients and supplements, such as SCFAs and vitamins (Bishehsari et al., 2018; Li et al., 2020), (3) detoxification of ingested dietary toxicants (Letertre et al., 2020), (4) modulation of host immune system function (Takiishi et al., 2017), and (5) gut–brain communication (Morais et al., 2021).

The characteristics of a healthy gut microbiome can be summed up in four words, namely, diversity, stability, resistance, and resilience. These four words correspond to the richness within the microbiota, the ability to adapt to environmental changes, and the ability of the microbiota to recover (Lozupone et al., 2012). To investigate the impact of gut microbes on humans, methods such as 16S ribosomal RNA gene sequencing (Johnson et al., 2019), metagenomic sequencing (Riesenfeld et al., 2004), and Illumina genome analyzers (Qin et al., 2010) are used to explore the composition of the microbiota. Alpha diversity (single site, such as the human gut) and beta diversity (multiple sites, such as different parts of the human body) were applied to evaluate sequencing data, and the human gut microbiota could be roughly divided into four phyla, namely, Bacteroidetes, Firmicutes, Proteobacteria, and Actinobacteria (Arumugam et al., 2011; Greenhalgh et al., 2016; Binda et al., 2018). Firmicutes and Bacteroidetes accounted for 90% of the intestinal flora abundance, and Proteobacteria and Actinobacteria accounted for 10%. The abundance of Bacteroidetes is associated with a high-fat, high-protein diet, and malnutrition (Johnson et al., 2017), while Firmicutes is increased in obese mice (Turnbaugh et al., 2006).

In healthy people, the composition of the gut microbiome is all about the same, but there is a marked difference in the microbial structure compared with epilepsy patients. According to Peng et al. (2018), an abnormally increased abundance of rare flora was observed in the patients with drug-resistant epilepsy, while patients with drug-sensitive epilepsy have an intestinal flora composition similar to healthy controls. Recently, a 22-year-old patient with drug-resistant epilepsy who received gut microbiota transplantation from a healthy individual remained seizure-free for 20 months despite discontinuation of anti-seizure medications (ASMs) (He et al., 2017). Therefore, it is inferred that normal gut microbiota can control epileptic seizures.

### Effects of Modulating Gut Microbiota on Epileptic Seizures

Diet is the most important factor in modifying the structure and function of intestinal flora (Zmora et al., 2019). The ketogenic diet (KD) and the modified Atkins diet are commonly used for epilepsy control. They are characterized by high fat and low carbohydrates, making the body switch from consuming glucose to consuming ketone bodies produced by fat metabolism as an energy source (Ułamek-Kozioł et al., 2019). A study shows that children with drug-resistant epilepsy treated with a KD for seizures had lower gut microbial diversity than healthy infants (Xie et al., 2017). The KD also had different effects on the intestinal microbiota composition of healthy infants and children with drug-resistant epilepsy. Firmicutes did not change significantly in healthy infants before and after KD treatment, but the proportion of Bacteroidetes increased. In addition, Proteobacteria was more enriched in children with drug-resistant epilepsy, and the proportion decreased after KD treatment (Xie et al., 2017). In the study by Wells et al. (2020), KD had a good effect on seizure control. This study has shown that elevated levels of ketone bodies (through a KD, exogenous ketonic supplements, or weight loss) can reduce airway hyperresponsiveness in obese asthmatic rats, which may be related to the inhibition of inflammatory responses (Mank et al., 2022).

The possible mechanism of the KD for epilepsy is not fully understood, but it may exert antiepileptic effects by increasing endogenous adenosine (Masino et al., 2009), opening ATP-sensitive potassium channels (Juge et al., 2010), acting on adenosine A1 receptors (Kadowaki et al., 2017), and inhibiting lactate dehydrogenase (Sada et al., 2015). Furthermore, according to Braakman and van Ingen (2018), six drug-resistant epilepsy patients acquired seizure-freedom during antibiotic use, suggesting that antibiotics may be a potential treatment for epilepsy.

Recently, probiotics represented by Bifidobacteria and Lactobacilli exist in our daily diets, such as yogurt, nutritional supplements, and fermented biscuits. One study showed that after oral administration of 8 mixed probiotics (mainly Lactobacillus) as a supplement to antiepileptic drugs in epilepsy patients, 28.9% of patients had more than 50% reduction in seizures, which was similar to other new ASMs; meanwhile, the quality of life (QoL) in the effective group of probiotics was significantly improved (Gómez-Eguílaz et al., 2018), and it is suggested that probiotic supplementation therapy may be a new method to control epilepsy.

## Possible Mechanisms by Which Innate Immunity Is Involved in Epileptic Seizures

The innate immune system is an evolutionarily ancient part consisting of barriers, small molecules, and cellular components. In 1885, Paul Ehrlich found that intravenous acid dyes could not stain brain tissue, and Emil Goldmann also found that brain tissue failed to stain after intravenous trypan blue in 1908 (Pachter et al., 2003), leading to the concept of BBB. The innate immune system protects organs and tissues from pathogenic damage without the need for additional measures (McComb et al., 2019). Innate immunity in the CNS includes the BBB, glial cells, and various cytokines.

### Blood–Brain Barrier

The BBB acts as the dividing line between the central and peripheral parts, preventing blood cells, pathogens, and poisons from entering the brain (Montagne et al., 2017). The phenomenon that neurons regulate the BBB function puts forward the neurovascular unit (NVU) concept. NVU is formed by the mutual coupling of vascular-related cells, glial cells, and neurons (Zlokovic, 2011), which regulates not only cerebral blood flow (Lecrux and Hamel, 2011) but also the tight junction (TJ) protein between endothelium can limit the paracellular permeability of the BBB, reducing the substance transport between cerebrospinal fluid and blood (Zlokovic, 2008), thereby maintaining central environmental homeostasis. The basis of this barrier function depends on the structure of TJs between endothelial cells, which are mainly composed of claudin, occludin, and ZO proteins (Berndt et al., 2019; Lochhead et al., 2020; Yuan et al., 2020). Among them, claudin-5 is the main protein constituting TJs (Greene et al., 2019). Research shows that the CNS of claudin-5 knockout mice displays higher permeability to macromolecules (Lochhead et al., 2020). At the same time, in cultured cerebral vascular endothelial cells, claudin-5 overexpression showed decreased paracellular permeability and increased tightness (Ohtsuki and Terasaki, 2007). The study by Li et al. (2016) showed that sodium butyrate could improve the neurological deficit after traumatic brain injury, upregulate the expression of TJs, and reduce the permeability of the BBB. Some studies have also pointed out that the local leakage of plasma proteins caused by the increased permeability of the BBB may play a critical role in epileptogenesis (Bankstahl et al., 2018).

### Gliacytes

In the CNS, astrocytes are one of the most abundant glial cells. They have an important role in the CNS and actively participate in the composition of the BBB or promote or limit the development of diseases (Linnerbauer et al., 2020). Furthermore, the role of astrocytes in epileptogenesis is increasingly well understood (Ivens et al., 2007; Seifert et al., 2010; Heinemann et al., 2012).

Glutamine synthase is an enzyme specifically expressed in astrocytes in the CNS, which can catalyze the synthesis of glutamine from ammonia and glutamate (Rose et al., 2013), thus maintaining CNS homeostasis, but decreased or lost glutamine synthase expression was found in patients with temporal lobe epilepsy (Sandhu et al., 2021), and this may be one of the possible mechanisms for inducing seizures. In addition, astrocytes also express a variety of potassium ion channels on the surface of astrocytes (Seifert et al., 2018) to maintain intracellular and extracellular potassium balance through potassium ion buffering and potassium ion uptake (Seifert and Steinhäuser, 2013). During the neuronal activity, extracellular potassium concentrations can rapidly fluctuate to upper levels, sufficient to induce seizures if the hyperkalemic environment is not properly corrected. Niday and Tzingounis (2018) suggested that extracellular potassium rises to depolarize neurons in the absence of Kir 4.1 channels, leading to the inactivation of sodium channels, thereby prolonging neuronal firing time or increasing the frequency, which causes neuronal damage hyperexcitability. Furthermore, in the research of Snowball et al. (2019), using a lentiviral vector packaging the engineered potassium channel (EKC) gene significantly reduced the number of seizures in focal neocortical epilepsy.

Microglia are macrophages in the brain parenchyma that derive from primitive hematopoiesis in the yolk sac (Aguzzi et al., 2013) and play an important role in neuronal health, apoptosis, and synapse formation (Nayak et al., 2014). Many studies show that microglia are closely related to neurological diseases (Hansen et al., 2018; Ho, 2019; Jia et al., 2021). The possible mechanisms include inflammation, gliosis, and stress. Microglia-derived inflammation is important in epileptogenesis, and ASMs with antiglial inflammatory properties benefit seizure control in a previous study (Dambach et al., 2014). Studies have also focused on the connection between microglia and neurons as the main factor for microglia activation, and the inflammatory proteins released by glial activation can increase excitability and contribute to epilepsy (Alyu and Dikmen, 2017).

### Cytokines

As part of innate immunity, cytokines are also closely related to the BBB. For example, in Qin et al.’s (2019) study, it was found that interleukin-1β can induce the pericyte NF-κB/p65 pathway leading to upregulation of matrix metalloproteinase-9 expression to the destruction of vascular endothelial TJs, thereby increasing the permeability of the BBB. TGF-β plays a crucial role in human development. When TGF-β binds to TGF-βR2, downstream SMAD proteins are phosphorylated and translocated to the nucleus, ultimately regulating the transcription of target genes of TGF-β (Vander Ark et al., 2018). Albumin present in brain tissue is translocated into cells by astrocytes through binding to TGF-β receptors on astrocytes and by downregulating the Kir 4.1 potassium channel on the surface of astrocytes, and it leads to the decrease of extracellular buffer potassium, increasing *N*-methyl-D-aspartate (NMDA) receptor-mediated neuronal excitability (Ivens et al., 2007).

The IL-1 family consists of pro- and anti-inflammatory cytokines (Yazdi and Ghoreschi, 2016). Among them, interleukin-1β is the most characteristic pro-inflammatory interleukin. Description of IL-1β was initially thought to be an endogenous pyrogen that induces the expression of COX-2, iNOS, TNF-α, IL-6, chemokines, adhesion molecules, and matrix metalloproteinases (Dinarello, 2005).

Studies have shown that the genes for IL-1β, IL-1R1, and IL-1RA are overexpressed in rodent models of epilepsy (Mukhtar, 2020). IL-1R1 is a receptor for IL-1β, belonging to the Toll-like/IL-1 receptor, activating prostaglandins (PGs) and NF-κB through MyD88 protein, thereby inducing inflammation (Yazdi and Ghoreschi, 2016). IL-1RA is an endogenous inhibitor of IL-1R1 (Ferrara-Bowens et al., 2017). Vezzani et al. (2008) suggested that IL-1β activates IL-1R1 on neurons and induces tyrosine phosphorylation of the NR2B subunit of NMDA receptor through Src kinase, leading to increased NMDA receptor-mediated calcium influx, thereby enhancing neuronal excitability. IL-1β also promotes glutamate release and inhibits glutamate reuptake by astrocytes through TNF-α, thereby inducing epileptiform events (Vezzani et al., 2008). IL-6 is a cytokine with dual effects, and proper IL-6 expression is very important for host defense function. After infection or injury, IL-6 is rapidly secreted and produced by monocytes/macrophages (Tanaka et al., 2016). In the healthy CNS, IL-6 is lowly expressed, but astrocytes and microglia become important sources of IL-6 in the CNS (Gruol, 2015). When TGF-beta on astrocytes can transmit information to the cells, upregulation of IL-6 leads to increased cortical excitability and finally induces epileptiform discharges *in vitro* (Levy et al., 2015).

Cyclooxygenase (COX) is an enzyme present on the cell membrane that catalyzes arachidonic acid to PGs (Clària, 2003). COX has three isoenzymes, of which COX-2 is an inducible enzyme associated with inflammation (Zhu et al., 2020), and found the induction of COX-2 in a hippocampal kindled rat model, suggesting that COX-2 is a key factor in epileptogenesis (Rawat et al., 2019). Rojas et al., 2014. found that celecoxib has an antiepileptic effect on acute seizures, but the antiepileptic effect of NSAIDs appears to be related to the time of administration (Rojas et al., 2014). Besides, a previous study by Kovács et al. (2011) showed that intraperitoneal injection of LPS in WAG/Rij rats enhanced their spike-wave discharge (Coenen and Van Luijtelaar, 2003), they subsequently found that the number and duration of spike-wave discharge increased after intraventricular injection of LPS in WAG/Rij rats, NSAIDs (indomethacin) could eliminate this phenomenon, and this strongly validates the role of COX in epilepsy.

HMGB1 is a highly conserved and proteinaceous structure in cells and acts as a classic alarm protein due to its ability to activate DAMP receptors of the innate immune system when present extracellularly (Yang et al., 2020). There are many reported receptors of HMGB1, such as RAGE, TLR9, TLR4, CD24, and CXCR4 (Paudel et al., 2018), but only RAGE and TLR4 are not controversial (Andersson et al., 2018). Recently, the role of HMGB1 in epilepsy has gradually attracted researchers’ attention. Zhao et al. (2017) showed that anti-HMGB1 mAbs could antagonize seizures in various epilepsy models and TLR4 knockout mice. This antiepileptic effect was absent (Zhao et al., 2017). Fu et al. (2017) also found that anti-HMGB1 monoclonal antibodies can delay HMGB1 translocation and downregulate the expression of inflammation-related inflammation-related factors.

Lipopolysaccharide, albumin, and SCFAs in the peripheral circulation can act on various receptors, resulting in increased calcium conductance, downregulated potassium channels, promotion of intracellular transcription, and release of immune molecules to cause BBB damage, which in turn lowers the seizure threshold or promotes epileptiform discharges, which eventually lead to seizures.

## The Possible Mechanism of Gut Microbiota Regulating Innate Immunity and Participating in Epilepsy

For the study of epileptogenesis, Devinsky et al. (2018) suggested that the spontaneous epileptic rat, which mimics the characteristics of human epileptic seizures, could be used as a research model for acquired epilepsy models, postnatal brain injury, or infection. In contrast, genetic models have spontaneous or induced genetic modifications that induce seizures. Epileptogenesis is caused by epileptogenic events (or risk factors) or genetic alterations that can persist long before the first clinical seizure and increase susceptibility to epilepsy, leading to spontaneous recurrent seizures (Pitkänen et al., 2015). Since the last century, the role of inflammation in epileptogenesis has been paid more and more attention. Inflammation is a defense mechanism of the body against damaging factors, and although the brain is considered an immune-privileged area, both innate and acquired immune responses can be rapidly induced in the CNS (Mukhtar, 2020).

As mentioned earlier, gut microbial structural stability plays an important role in metabolism, immunity, and homeostasis. Disturbances in the microbial structure are associated with a variety of neurological diseases, such as autism (Saurman et al., 2020), PD (Elfil et al., 2020), AD (Sochocka et al., 2019), and even affect mental behavior (Lach et al., 2018; Peirce and Alvi na, 2019). More and more studies have linked epilepsy susceptibility to changes in gut microbiota structure. For example, probiotic supplementation can reduce seizures by more than 50% in patients with epilepsy (Gómez-Eguílaz et al., 2018), antibiotics can affect seizures (Ghanizadeh and Berk, 2015; Lum et al., 2020), and dietary therapy can alter the structure of intestinal microbes to reduce seizures (Zhang et al., 2018). Another study found that mice transplanted with gut microbiota from depressed patients developed depression-like behaviors, while mice transplanted with gut microbiota from healthy individuals did not develop depression-like behaviors (Zheng et al., 2016).

Gut microbes can also affect CNS function, and Goehler et al. (2005) also found in mice fed specific strains that gut microbes can transmit pathogen signals to the CNS *via* the vagus nerve. Some pathogens in the gut can produce toxins (such as LPS) that cross the intestinal mucosal barrier and then enter the circulation, and finally, cross the BBB to induce neuroinflammation (Alexandrov et al., 2019). Neuroinflammation is well documented in AD and PD (Heneka et al., 2015; Rocha et al., 2018; Megur et al., 2020; Leng and Edison, 2021). Of course, it also contributes to epileptogenesis (Paudel et al., 2018; Sharma et al., 2019; Hodges and Lugo, 2020). In the experiments of Hu et al. (2020), the alteration of gut microbiota induced by a high-salt diet led to a decrease in the production of gut-derived SCFA and induced BBB dysfunction and microglial activation in mice, as well as the expression of cortical IL-1β, IL-6, and TNF-α.

Lipopolysaccharide is present on the surface of many bacterial membranes, and the host’s immune system can trigger the immune response by recognizing the conserved structures of microbial species in a manner called PAMPs (Takeuchi and Akira, 2010; Bertani and Ruiz, 2018). Gao et al. (2014) found elevated concentrations of TNF-α and IL-1β in hippocampal slices exposed to LPS early and that LPS could increase the frequency of epileptiform discharges and neuronal excitability. Györffy et al. (2014) suggested that in a genetic absence epilepsy model, WAG/Rij rat, peripheral injection of LPS can promote spike-wave discharge and found a variety of differentially expressed proteins in the brain, most of which are associated with epilepsy, LPS-related inflammation, and sleep. The experiments of Brunner et al. (2021) confirmed that exogenous supplementation of ketogenic supplement in WAG/Rij rats can reduce LPS-induced spike-wave discharge by inhibiting the inflammatory response.

As a significant neurotoxin and pro-inflammatory substance, LPS is a typical ligand of TLR4, which can alter synaptic transmission and affect long-term potentiation (Vezzani et al., 2013). In addition, the intracerebral injection of LPS lowered the seizure threshold (Matin et al., 2015). The use of LPS in the rat cerebral cortex can induce increased neuronal excitability and produce epileptiform discharges, and this effect can be antagonized by IL-1RA, suggesting that LPS may exert such effects through IL-1R (Vezzani et al., 2013). Studies have also shown that intestinal inflammation can increase susceptibility to epilepsy and reduce the efficacy of ASMs (De Caro et al., 2019). Riazi et al. (2010) found that intestinal inflammation exacerbates seizures, and inducing peripheral inflammation using LPS increases epilepsy susceptibility. Endogenous ligands of TLR4, including HMGB1 and IL-1β, are produced by glial cells after brain injury, thereby mimicking LPS to exert pro-inflammatory effects (Devinsky et al., 2013).

The BBB is the major regulator of various molecules and cells into or out of the CNS, including microglia, endothelial cells, astrocytes, pericytes, and basement membranes (Sharif et al., 2018). TJs between endothelium are a major factor in determining their permeability, and a lack of TJ protein expression shows increased permeability to small molecules (Sweeney et al., 2019). LPS downregulates the expression of TJs, thereby increasing the permeability of the BBB. The possible mechanisms include the destruction of the BBB caused by PGs or nitric oxide (Varatharaj and Galea, 2017). Other possible factors include matrix metalloproteinases (Qin et al., 2015) and reactive oxygen species (Yu et al., 2015). The permeability of the BBB can also be modulated by intestinal flora, and microbial-derived metabolites can promote the integrity of the BBB (Parker et al., 2020), which controls the flow of circulating substances into and out of the brain. Braniste et al. (2014) suggested that intestinal flora disturbance is beneficial to increasing BBB permeability. The most pronounced change following increased BBB permeability is albumin penetration (van Vliet et al., 2015), which is involved in epileptogenicity by inducing excitatory synaptogenesis by binding to TGF-β receptors on astrocytes (Weissberg et al., 2015). In addition, albumin binding to TGF-β induces seizures by mediating impaired extracellular potassium buffering and increased neuronal excitability (Ivens et al., 2007).

Furthermore, LPS-induced systemic inflammation can lead to transcriptional activation of microglia inflammatory genes throughout the brain (Soulet and Rivest, 2008). After the IL-1R1 and TLR4 expressed on its surface are activated by endogenous ligands, the transcription of inflammatory genes mediated by NF-κB and activator protein-1 (AP-1) is formed, thereby continuing the inflammatory event (Vezzani et al., 2016). Microglia, as the primary immune cells in the brain, participate in the formation of NVU (Abbott et al., 2010) and can respond rapidly to any damage to the CNS (Liu et al., 2020), and their pro-inflammatory phenotype (M1) has demonstrated a damaging effect on the NVU (Boche et al., 2013). In the study by Ronaldson and Davis (2020), after 7 days of *in vivo* injection of LPS to induce inflammation, it was found that activated microglia can damage the integrity of the BBB and cause BBB leakage, to understand the effect of circulating cytokines on the BBB; Nzou et al. (2020) co-incubated tissue sections with exogenous cytokines and found that IL-6 and TNF-α can also affect the distribution of TJs, leading to leakage of the BBB. This evidence suggests that systemic inflammation has a role in regulating the permeability of the BBB. Mazarati et al. (2017) also suggested that inflammatory cytokines produced by peripheral inflammation activate microglia through peripheral afferent nerves or the BBB, thereby synthesizing cytokines to induce inflammation.

## Conclusion

**FIGURE 1:**
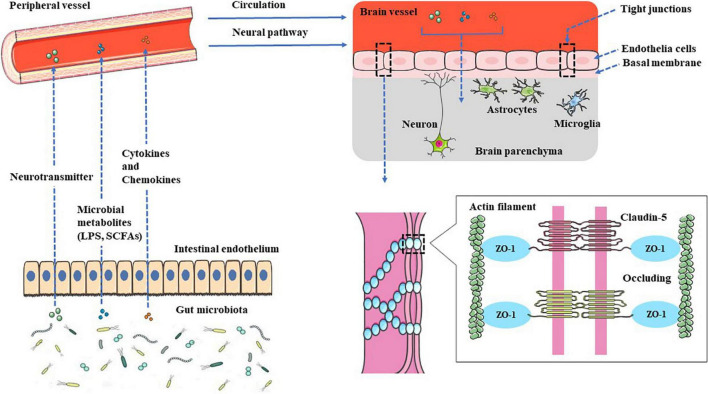
Gut–brain communication. Gut microbial surface substances and their metabolites can positively or negatively affect the CNS through the peripheral circulation or enteric nervous system, and microbial-derived neurotransmitters have regulatory effects on neurons or nerve cells after entering the brain. The metabolites can not only play an anti-inflammatory effect, protect the BBB but also play a pro-inflammatory effect, activate microglia to secrete inflammatory mediators, and then damage the BBB, leading to leakage.

**FIGURE 2:**
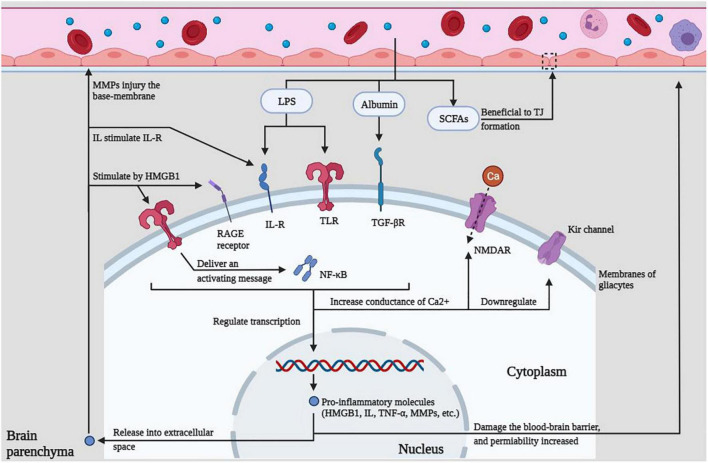
Innate immune regulation within the CNS. LPS, albumin, and SCFAs in the peripheral circulation can act on various receptors, resulting in increased calcium conductance, down-regulated potassium channels, promotion of intracellular transcription, and release of immune molecules to cause BBB damage, which in turn lowers the seizure threshold or promotes epileptiform discharges, which eventually lead to seizures.

The relationship between epilepsy and gut microbes has received more and more attention, and related research is also in the ascendant. The intestine is the largest gathering place of microorganisms in the human body. A reasonable microbial population can promote the formation of the intestinal immune system and become the immune barrier of the human body. The disorder of the intestinal flora structure weakens the function of the intestinal immune barrier. The various innate immune molecules produced by it can induce local or systemic inflammation and even cause damage to the distant BBB structure through its surface molecules or metabolites (see Figure 1). The destruction of the BBB exposes the brain tissue to the attack of peripheral inflammatory factors or immune cells, which in turn induces glial cells in the brain to produce inflammatory factors, lowers the seizure threshold or induces epileptiform discharges, and even directly leads to epileptic seizures (see Figure 2), all of these have positive effects on the occurrence and development of epilepsy. This study reviews the possible mechanisms by which gut microbes regulate innate immune function and participate in the development of epilepsy, providing a better direction for further research in this field.

## Author Contributions

LZ, SL, and ZT designed and wrote the manuscript. ZX and CY helped with proofreading and revision. All authors contributed to the article and approved the final version.

## Conflict of Interest

The authors declare that the research was conducted in the absence of any commercial or financial relationships that could be construed as a potential conflict of interest.

## Publisher’s Note

All claims expressed in this article are solely those of the authors and do not necessarily represent those of their affiliated organizations, or those of the publisher, the editors and the reviewers. Any product that may be evaluated in this article, or claim that may be made by its manufacturer, is not guaranteed or endorsed by the publisher.
